# Impact of emphysema on sputum culture conversion in male patients with pulmonary tuberculosis: a retrospective analysis

**DOI:** 10.1186/s12890-020-01325-1

**Published:** 2020-11-07

**Authors:** Naoki Takasaka, Yoshitaka Seki, Ikumi Fujisaki, Shota Uchiyama, Sachi Matsubayashi, Akihito Sato, Yumie Yamanaka, Kyuto Odashima, Taisuke Kazuyori, Aya Seki, Hiroshi Takeda, Takeo Ishikawa, Kazuyoshi Kuwano

**Affiliations:** 1 Department of Internal Medicine, Division of Respiratory Diseases, The Jikei University Daisan Hospital, 4-11-1 Izumihoncho Komae-shi, Tokyo, 201-8601 Japan; 2 Department of Internal Medicine, Division of Respiratory Diseases, The Jikei University School of Medicine, Tokyo, Japan

**Keywords:** Tuberculosis, Emphysema, Culture conversion

## Abstract

**Background:**

Although cigarette smoking may have a negative impact on the clinical outcome of pulmonary tuberculosis (PTB), few studies have investigated the impact of smoking-associated lung diseases. Emphysema is a major pathological finding of smoking-related lung damage. We aimed to clarify the effect of emphysema on sputum culture conversion rate for *Mycobacterium tuberculosis* (MTB).

**Methods:**

We retrospectively studied 79 male patients with PTB confirmed by acid-fast bacillus smear and culture at Jikei University Daisan Hospital between January 2015 and December 2018. We investigated the sputum culture conversion rates for MTB after starting standard anti-TB treatment in patients with or without emphysema. Emphysema was defined as Goddard score ≥ 1 based on low attenuation area < − 950 Hounsfield Unit (HU) using computed tomography (CT). We also evaluated the effect on PTB-related CT findings prior to anti-TB treatment.

**Results:**

Mycobacterial median time to culture conversion (TCC) in 38 PTB patients with emphysema was 52.0 days [interquartile range (IQR) 29.0–66.0 days], which was significantly delayed compared with that in 41 patients without emphysema (28.0 days, IQR 14.0–42.0 days) (*p* < 0.001, log-rank test). Multivariate Cox proportional hazards analysis showed that the following were associated with delayed TCC: emphysema [hazard ratio (HR): 2.43; 95% confidence interval (CI): 1.18–4.97; *p* = 0.015), cavities (HR: 2.15; 95% CI: 1.83–3.89; *p* = 0.012) and baseline time to TB detection within 2 weeks (HR: 2.95; 95% CI: 1.64–5.31; *p* < 0.0001). Cavities and consolidation were more often identified by CT in PTB patients with than without emphysema (71.05% vs 43.90%; *p* = 0.015, and 84.21% vs 60.98%; *p* = 0.021, respectively).

**Conclusions:**

This study suggests that emphysema poses an increased risk of delayed TCC in PTB. Emphysema detection by CT might be a useful method for prediction of the duration of PTB treatment required for sputum negative conversion.

## Background

About 10 million people were newly diagnosed with active tuberculosis (TB) and 1.2 million patients died in 2018 [1]. TB clinical features are complicated by bacterial aspects, including drug-resistant *Mycobacterium tuberculosis* (MTB), and impaired host immune response caused by human immunodeficiency virus (HIV), immunosuppressive therapy, or malignancy [2]. Sputum culture conversion is widely used to determine bacteriological response to anti-TB therapy and is associated with long-term cure [3]. According to recent reports, cigarette smoking is related to delayed culture conversion and treatment failure [4–6]. However, the relationship between actual smoking-related lung damage and pulmonary TB (PTB) is still unknown. Emphysema is a major pathological feature in chronic obstructive pulmonary disease (COPD). The most important aspects of emphysema are sustained oxidative stress and cell damage mediated by macrophages and other cells of the innate and adaptive immune systems, leading to epithelial cell death [7]. Macrophages are localized to sites of alveolar wall destruction in patients with emphysema, and secrete many inflammatory cytokines that contribute to smoking-induced emphysema via elastosis [8]. As a result, there is a correlation between macrophage numbers and severity of emphysema [9]. Alveolar macrophages (AMs) from patients with COPD show reduced phagocytic uptake of bacteria, which might be a factor in bacterial colonization [10].

We have focused on detection of emphysema by computed tomography (CT). Recently, CT biomarkers for emphysema have revealed the relationship between emphysema and COPD phenotype, including airflow limitation and elastic enzyme release [11–14]. Therefore, we hypothesized that CT-detected emphysema might have a negative effect on clinical features of PTB, such as smoking-induced immune dysfunction. To clarify the impact of CT-detected emphysema on culture conversion rates in PTB patients, we performed a retrospective study in our hospital between 2015 and 2018. We showed that CT-detected emphysema had a negative impact on sputum culture conversion in PTB patients.

## Methods

### Study design

In this retrospective study, we analysed data from hospitalized patients with positive acid-fast bacillus (AFB) smears at Jikei University Daisan Hospital between January 2015 and December 2018. During hospitalization, patients maintained smoking cessation. Exclusion criteria were: (1) age < 40 years; (2) isolation of MTB resistant to isoniazid (INH) or rifampicin (RMP); (3) patients not receiving standard anti-TB regimens with INH, RMP, ethambutol (EMB) and pyrazinamide, or INH, RMP and EMB; (4) patients required desensitization to any adverse effects or comorbidities; (5) co-infection with non-TB mycobacteria; (6) death within 28 days after anti-TB treatment initiation because sputum MTB conversion could not be confirmed; and (7) female sex because only three female PTB patients had emphysema. Sputum smear and culture examinations were performed at the start of treatment and then every 2 weeks, with a margin of error of a few days, during hospitalization. The sputum AFB smear was examined using a fluorochrome procedure according to the American Thoracic Society guidelines [15]. Sputum specimens were cultured in liquid (Mycobacterial Growth Indicator Tubes, MGIT; BD, Franklin Lakes, NJ, USA). Time to sputum culture conversion (TCC) was defined as the number of days between treatment initiation and the first negative sputum culture and no positive culture thereafter.^7^ The delayed conversion may be explained by the high baseline bacillary load. Baseline time to TB detection (TTD) was defined as time (in days) until MGIT culture showed a positive result at initial sputum culture [6, 16]. Atwine et al. reported a twofold increase in risk of culture conversion at 2 months in patients with baseline TTD < 14 days compared with ≥14 days [17]. This study was approved by the Ethics Committee of Jikei University School of Medicine [No. 30–003(9024)].

### Radiological classification

Baseline chest high-resolution CT (HRCT) was performed on all patients. HRCT was performed using a helical CT scanner (SOMATOM Definition AS+; Siemens, Erlangen, Germany) with the following parameters: 1.0–5.0-mm slice thickness, 0.3-mm slice separation, 120-kV tube voltage, 140 mAs, 0.6-mm collimation, 0.5-s rotation time, and 0.85 beam pitch. Images reconstructed with the B60f kernel were used for image analysis. CT scans were requested to establish diagnosis and evaluate active PTB lesions before treatment.

TB-related CT findings were evaluated by considering consolidation, nodule/mass, bronchiolitis and cavities in both lungs [18, 19]. Consolidation was defined as homogeneous increased lung attenuation that obscured the margin of vessels and airways. A nodule/mass was defined as a well-demarcated spherical area of soft tissue attenuation, ≤3 cm in diameter. Bronchiolitis was defined as the presence of centrilobular micronodules (< 10 mm in diameter) and branching nodular structures (including tree-in-bud appearance). Cavities were defined as round, gas- or fluid-filled spaces within a nodule/mass or consolidation. CT-detected emphysema was defined as low attenuation area (LAA) < − 950 HU, (LAA_<-950_) [11, 20]. Emphysema severity was assessed according to the Goddard Scoring System [21, 22].: 0, normal; 1, LAA_<-950_ ≤ 25% affected; 2, > 25% and ≤ 50% affected; 3, > 50% and ≤ 75% affected; and 4, > 75% affected. Six images were analyzed in three slices, which were obtained from the aortic arch level, carina level, and 1 cm above the right diaphragm, and a total score for six images was calculated for each patient. Two pulmonologists with 11 and 16 years’ experience interpreted chest CT results. When there were discrepancies in their readings, final decisions were reached by consensus. Goddard score was determined as the average value scored by the pulmonologists. Total Goddard score ≥ 1 was defined as emphysema, as reported previously [22], because 95% of nonsmokers had lungs with < 5% emphysematous involvement [23].

### Statistical analysis

Statistical analysis was performed using IBM SPSS statistics version 24.0. (IBM Corp., Armonk, NY, USA). Student’s *t*-test or Fischer’s exact test was used to compare the distributions of characteristics between PTB patients with and without emphysema. Cox proportional hazards regression analysis was used to estimate the hazard ratio (HR) of sputum culture conversion for the following prespecified baseline variables: age, emphysema, smoking history, presence of consolidation, cavity lesions, hypoalbuminemia, and baseline TTD within 2 weeks. HRs were calculated with 95% confidence intervals (CIs). A *p* value < 0.05 indicated statistical significance in all analyses. Kaplan–Meier curves for TB sputum conversion probability were plotted for patients with emphysema versus those without emphysema. The log-rank test was used to test the significance in subgroups of patients with emphysema.

## Results

### Patient characteristics

**Fig. 1:**
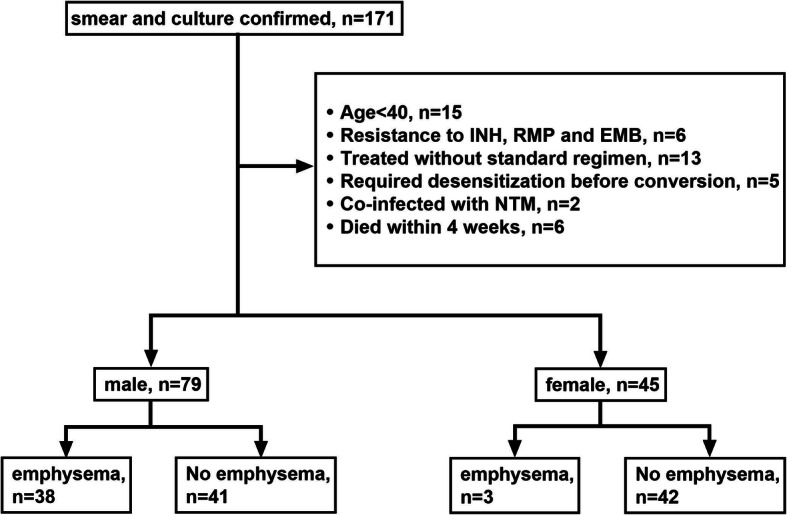
Flow chart of patient selection. EMB, ethambutol; INH, isoniazid; NTM, nontuberculous mycobacteria; RMP, rifampicin

**Fig. 2:**
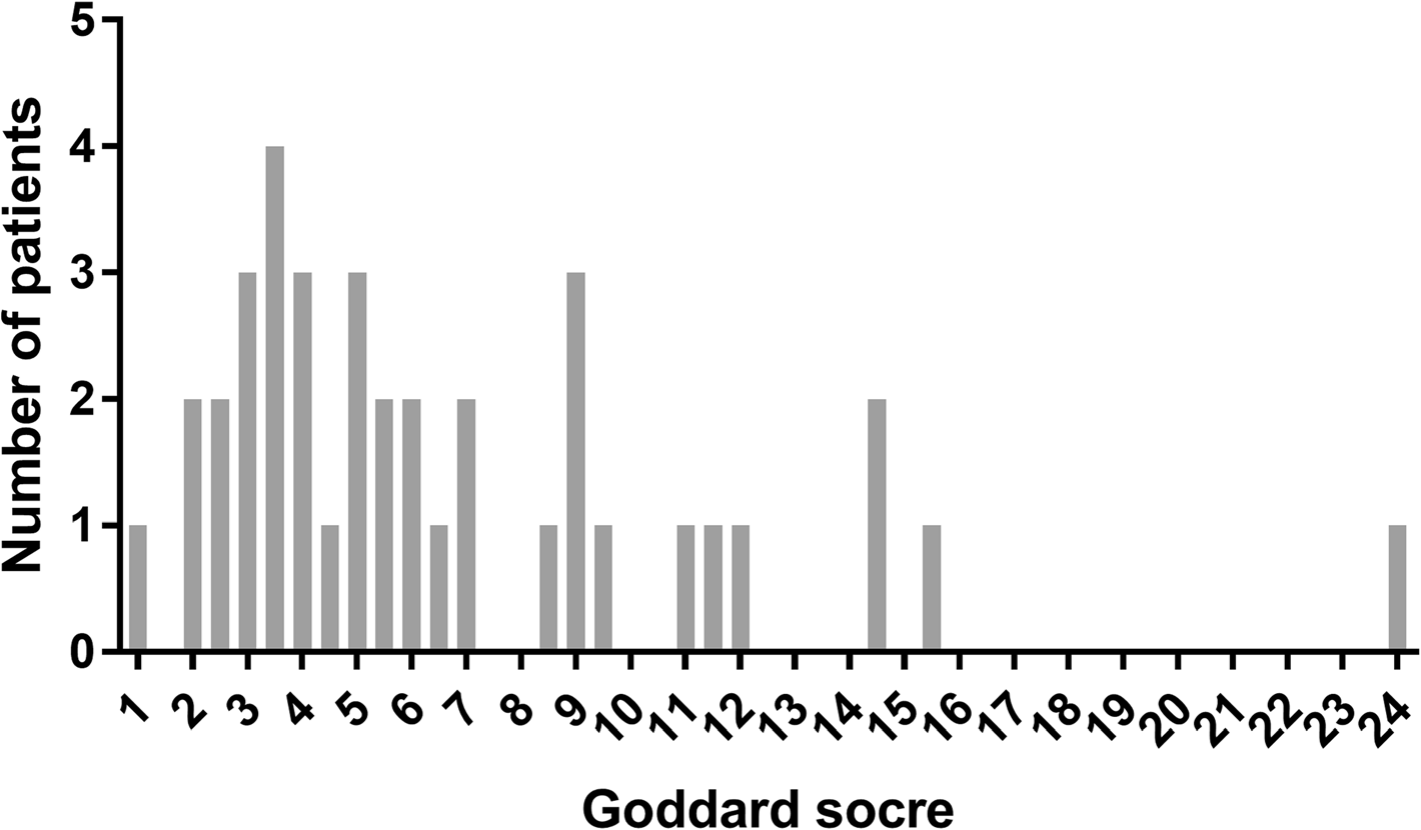
The distribution of Goddard score in the pulmonary tuberculosis patients with emphysema (n = 38)

Between January 2015 and December 2018, 171 patients were diagnosed and hospitalized with active TB with AFB smear- and culture-positive sputum at Jikei Daisan Hospital (Fig. 1). Ninety-two patients were excluded according to the exclusion criteria. The mean age was 69.01 ± 15.92 years. No patient was positive for HIV antibody. There was no significant difference in mean age, proportion of patients with chronic kidney disease or diabetes, or baseline TTD between patients with or without emphysema (Table 1). PTB patients with emphysema had a significantly higher proportion with smoking history (100% vs 41.46%, *p* < 0.0001), current smoking habit (52.63% vs 14.63%, *p* < 0.001), and hypoalbuminemia (81.58% vs 46.34%, *p* = 0.001) (Table 1). About a quantitative assessment of emphysema, average Goddard score of emphysema patients was 6.74 ± 4.73 (Table 1, Fig. 2).

**Table 1:** Characteristics of patients with pulmonary tuberculosis with or without emphysema

Characteristics	Total (*n* = 79)	With emphysema (*n* = 38)	Without emphysema (*n* = 41)	*p-* value*
Demographics				
Age, years	69.01 ± 15.92	69.18 ± 16.35	68.85 ± 15.72	0.927
Smoking history	55 (69.62%)	38 (100%)	17 (41.46%)	< 0.0001
Current smoker	26 (32.91%)	20 (52.63%)	6 (14.63%)	< 0.001
Alcohol consumption	27 (34.18%)	17 (44.74%)	10 (24.39%)	0.057
AST (IU/L)	29.29 ± 25.14	33.13 ± 31.65	25.73 ± 16.69	0.193
ALT (IU/L)	21.58 ± 21.32	24.03 ± 26.06	19.32 ± 15.73	0.330
Total bilirubin (mg/dL)	0.55 ± 0.35	0.45 ± 0.20	0.64 ± 0.43	0.011
Diabetes^†^	22 (27.85%)	13 (34.21%)	9 (21.95%)	0.225
Haemoglobin A1c (%) (missing value = 1)	6.42 ± 1.50	6.40 ± 1.26	6.43 ± 1.71	0.930
CKD^‡^	19 (24.05%)	7 (18.42%)	12 (29.27%)	0.260
eGFR (mL/min/1.73 m^2^)	77.94 ± 40.36	79.37 ± 30.10	76.61 ± 48.33	0.764
Hypoalbuminemia^§^	50 (63.29%)	31 (81.58%)	19 (46.34%)	0.001
Albumin (g/dL)	3.14 ± 0.76	2.93 ± 0.68	3.33 ± 0.80	0.020
Autoimmunity	5 (6.33%)	1 (2.63%)	4 (9.76%)	0.361
Steroid use	8 (10.13%)	2 (5.26%)	6 (14.63%)	0.266
Malignancy	9 (11.39%)	4 (10.53%)	5 (12.20%)	1.0
CRP (mg/dL)	5.08 ± 4.35	5.58 ± 3.61	4.60 ± 4.95	0.320
TTD at baseline (days)	17.19 ± 7.07	17.13 ± 7.75	17.24 ± 6.47	0.944
TTD within 2 weeks at baseline	54 (68.35%)	25 (65.79%)	29 (70.73%)	0.637
Treated with HREZ	54 (68.35%)	27 (71.05%)	27 (65.85%)	0.620
Goddard score	3.24 ± 4.70	6.74 ± 4.73	0	< 0.0001

Data are means ± SD, n (%)

*We used Fisher’s exact test or Student’s t-test to calculate *p-* values

^†^Diabetes: prescription of oral hypoglycaemic agents and/or insulin; fasting blood glucose ≥126 mg/dL or haemoglobin A1c ≥6.5%. ^‡^ CKD: glomerular filtration rate < 60 mL/min/1.73 m2 for > 3 months. ^§^ Hypoalbuminemia: serum albumin ≤3.5 g/dL

*SD* standard deviation, *CKD* chronic kidney disease, *TTD* time to tuberculosis detection, *HREZ* isoniazid, rifampicin, ethambutol and pyrazinamide

### CT findings related to PTB

We compared PTB-related CT findings including cavities, consolidation, bronchiolitis, and nodule/mass between PTB patients with or without emphysema. Cavities and consolidation were seen more frequently in PTB patients with than without emphysema (71.05% vs 43.90%, *p* = 0.015, and 84.21% vs 60.98%; *p* = 0.021, respectively) (Table 2).

**Table 2:** CT findings in patients with pulmonary tuberculosis with or without emphysema

CT findings	Total n = 79	With emphysema ***n*** = 38	Without emphysema n = 41	***p***- value*
Cavities	45 (56.96%)	27 (71.05%)	18 (43.90%)	0.015
Consolidation	57 (72.15%)	32 (84.21%)	25 (60.98%)	0.021
Bronchiolitis	62 (78.48%)	28 (73.68%)	34 (82.93%)	0.318
Nodule/mass	29 (36.71%)	16 (42.11%)	13 (31.71%)	0.338

Data are n (%)

*We used Fisher’s exact test

### Sputum TCC

**Fig. 3:**
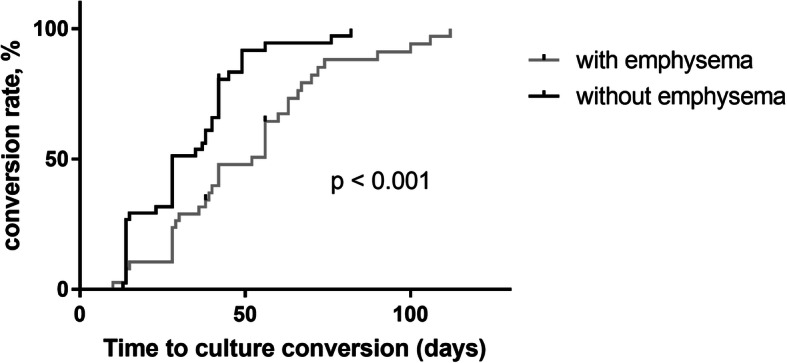
Proportion of pulmonary tuberculosis (PTB) patients with sputum culture conversion after treatment initiation (with vs without emphysema). Kaplan–Meier curves revealed significantly longer sputum time to culture conversion (TTC) in 38 PTB patients with emphysema (median TCC 52.0 days) than in 41 patients without emphysema (28.0 days) (p < 0.001, log-rank test)

In the Kaplan–Meier analysis, 38 PTB patients with emphysema needed a median TCC of 52.0 days [interquartile range (IQR) 29–66 days], which was significantly longer than 28.0 days (IQR 14–42 days) in 41 patients without emphysema (*p* < 0.001, log-rank test) (Fig. 3). Univariate Cox proportional hazards regression analysis showed that the following were associated with delayed TCC: emphysema (HR: 2.21; 95% CI: 1.36–3.60; *p* = 0.001); smoking history (HR: 1.81; 95% CI: 1.09–2.99; *p* = 0.021); consolidation (HR: 1.77; 95% CI: 1.06–2.96; *p* = 0.031); cavities (HR: 2.72; 95% CI: 1.65–4.49; *p* < 0.0001); hypoalbuminemia (HR: 1.74; 95% CI: 1.08–2.80; *p* = 0.024); and baseline TTD within 2 weeks (HR: 2.56; 95% CI: 1.52–4.31; *p* < 0.0001) (Table 3). Multivariate Cox proportional hazards regression analysis showed that the following were associated with delayed TCC after adjusting for age, emphysema, smoking history, cavities, consolidation, hypoalbuminemia, and baseline TTD within 2 weeks: emphysema (HR: 2.43; 95% CI: 1.18–4.97; *p* = 0.015); cavities (HR: 2.15; 95% CI: 1.18–3.89; *p* = 0.012); and baseline TTD within 2 weeks (HR: 2.95; 95% CI: 1.64–5.31; p < 0.0001) (Table 3).

**Table 3:** Cox proportional hazard regression unadjusted and adjusted analysis of time to sputum culture conversion*

Characteristics	Univariate HR (95% CI)	***p***- value	Multivariate HR (95% CI)		***p***- value
Age	1.01 (0.99–1.02)	0.483	0.994 (0.98–1.01)		0.593
Emphysema	2.21 (1.36–3.60)	0.001	2.43 (1.18–4.97)		0.015
Smoking history	1.81 (1.09–2.99)	0.021	0.87 (0.44–1.72)		0.692
Current smoker	1.26 (0.77–2.04)	0.361			
Consolidation	1.77 (1.06–2.96)	0.031	1.07 (0.61–1.87)		0.818
Cavities	2.72 (1.65–4.49)	< 0.0001	2.15 (1.18–3.89)		0.012
Alcohol consumption	1.19 (0.73–1.94)	0.478			
Diabetes^†^	1.67 (0.99–2.82)	0.055			
CKD^‡^	0.83 (0.48–1.41)	0.482			
Hypoalbuminemia^§^	1.74 (1.08–2.80)	0.024	0.99 (0.53–1.86)		0.979
Autoimmunity	0.44 (0.18–1.11)	0.081			
Steroid use	0.83 (0.38–1.82)	0.641			
Malignancy	1.02 (0.51–2.06)	0.956			
TTD within 2 weeks at baseline	2.56 (1.52–4.31)	< 0.0001	2.95 (1.64–5.31)		< 0.0001
Treated with HREZ	1.40 (0.85–2.31)	0.181			

*HR calculated with each per unit increase. Each variable measured at the time of diagnosis. Adjusted by previously described clinically important variables and candidate variables (age, emphysema, smoking history, consolidation, cavities, hypoalbuminemia and TTD within 2 weeks) for which the *p-* value was < 0.05 on univariate analysis

^†^Diabetes: prescription of oral hypoglycaemic agents and/or insulin; fasting blood glucose ≥126 mg/dL or haemoglobin A1c ≥6.5%. ^‡^ CKD: glomerular filtration rate < 60 mL/min/1.73 m2 for > 3 months. ^§^ Hypoalbuminemia: serum albumin ≤3.5 g/dL

## Discussion

We evaluated the impact of emphysema on AFB sputum TCC rates following initiation of anti-TB treatment in hospitalized PTB patients at Jikei University Daisan Hospital. Median TCC was significantly delayed in 38 PTB patients (52.0 days) with emphysema compared with 41 patients without emphysema (28.0 days). Multivariate Cox proportional hazards analysis showed that emphysema, cavities, and baseline TTD within 2 weeks were associated with delayed TCC. With regard to PTB-related CT findings, cavities and consolidation were seen more frequently in PTB patients with than without emphysema (71.05% vs 43.90%; *p* = 0.015, and 84.21% vs 60.98%; *p* = 0.021, respectively).

To our knowledge, this is the first study to report the impact of emphysema on sputum culture conversion in PTB patients. We focused on emphysema detection using CT. Emphysema was defined using the current standard of LAA_<-950_ [11, 14]. Although no well-validated biomarker of COPD has been identified other than forced expiratory volume in 1 s (FEV_1_), various quantitative imaging methods for emphysema are being developed to reveal the relationship between emphysema and COPD phenotype, including airflow limitation and level of matrix metalloproteinases (MMPs) [11–14]. CT is a minimally invasive technique that can identify the pathological features of emphysema. Goddard score based on the LAA proportion is widely used to measure emphysema [22, 24, 25]. Average Goddard score of emphysema patients was 6.74 ± 4.73 in our study. The severity of most emphysema cases was categorised as mild or moderate according to previous reports [26]. Our data suggest that even a mild level of emphysema has a negative influence on the duration of PTB treatment required for sputum culture conversion. Multivariate Cox proportional hazards analysis showed that emphysema was more significantly associated with delayed MTB culture conversion than smoking history was (not significant). According to recent reports, smoking history (current or former) has a negative impact on PTB clinical outcome, including delayed culture conversion [6, 27]. Although smoking history based on medical interview is a simple method to assess smoking exposure, the relationship between amount of smoking and smoking-associated immune response remains unclear. The reversibility of immune modification by smoking cessation is still unknown. Our results suggest that emphysema detection using CT is useful for predicting the duration of TB treatment required for sputum culture negative conversion.

The host immune response is crucial in TB pathogenesis, as demonstrated by the increase in incidence and mortality in immunocompromised hosts [2]. AMs are involved in the host inflammatory defence against MTB [28]. MTB is transmitted through air droplets and is an intracellular bacterium that infects AMs and dendritic cells (DCs) localized to the alveoli. The innate immune response is activated and inflammatory cells are recruited to the lungs. MTB that can evade the host immune system within AMs and DCs drives granulomatous inflammation and establishes PTB.

Recent reports suggest that smoking habit increases the risk of developing active TB and mortality [27, 29], and smoking-modulated immune dysfunction might be involved in TB pathogenesis [6, 27]. Although the pathogenesis of COPD/emphysema is not fully understood, it is associated with smoking-induced chronic inflammation of airways and lung parenchyma. After amplification of inflammation in patients with COPD, abnormal inflammatory immune responses are sustained even after smoking cessation [30]. AMs also play a central role in chronic inflammation in COPD [9]. AMs are activated by cigarette smoke extract, and secrete inflammatory mediators, including tumour necrosis factor-α, chemokine C-X-C motif ligand (CXCL)1 and CXCL8. AMs release elastic enzymes (MMPs) that contribute to emphysematous changes [31]. Macrophages are localized to sites of alveolar wall destruction in patients with emphysema, and there is a correlation between number of AMs and severity of emphysema [9]. AMs from patients with COPD show reduced phagocytic uptake of bacteria, which might be a factor in bacterial colonization [10, 29]. These reports suggest that smoking-associated immune modification in emphysema is involved in delayed TCC in terms of increased PTB severity and reduced response to anti-TB therapy.

Patients’ immune function also affects the radiological findings of PTB [32]. PTB-related CT findings differ between patients with or without emphysema. Jeon et al. reported that PTB patients with emphysema often show pneumonia-like consolidation, suggesting that impaired innate immunity in emphysema causes PTB progression and expansion of inflammation [18]. In our study, cavities and consolidation were seen more frequently in PTB patients with emphysema than in those without emphysema (71.05% vs 43.90%; *p* = 0.015, and 84.21% vs 60.98%; *p* = 0.021, respectively) (Table 2). Cavity lesions are associated with the greatest bacterial load, suggesting highly infectious and severe PTB [33, 34]. It is speculated that smoking-related immune modification in emphysema contributes to more severe radiological findings and extension of TCC.

This study had some limitations. First, this was a small, single-center retrospective study. Nevertheless, our finding of a negative effect of emphysema on sputum TCC suggests that emphysema is a potential risk factor for PTB development in terms of smoking-related immune dysfunction. In future, a larger prospective study could reveal the relationship between CT-detected emphysema and clinical outcome of PTB. Second, we needed to enroll patients with confirmed COPD with emphysema to determine the relationship between COPD/emphysema and sputum culture conversion. To diagnose COPD, airflow limitation must be identified with spirometry. However, it is impossible to estimate accurately airflow limitation in patients with COPD and active PTB at baseline.

Third, we could not analyze female patients because there were only three women with PTB with emphysema. From the 1970s to 1990s, smoking rate for Japanese women was only 10–15%, while that for men was 55–80% [35]. Although the difference became smaller after the 2000s, the risk of emphysema in Japanese women is potentially lower than in men.

## Conclusions

In conclusion, CT-detected emphysema with a Goddard score ≥ 1 is associated with delayed AFB sputum culture conversion and PTB-related CT findings. If confirmed in larger populations of PTB patients with emphysema, CT-detected emphysema could be helpful for predicting the duration of PTB treatment required for sputum culture negative conversion.

## Data Availability

The datasets used and/or analyses during the current study available from the corresponding author on reasonable request.

## Abbreviations

PTB: Pulmonary tuberculosis
MTB: *Mycobacterium tuberculosis*
TB: Tuberculosis
HU: Hounsfield Unit
CT: Computed tomography
TCC: Time to culture conversion
IQR: Interquartile range
HR: Hazard ratio
CI: Confidence interval
HIV: Human immunodeficiency virus
COPD: Chronic obstructive pulmonary disease
AM: Alveolar macrophage
AFB: Acid-fast bacillus
INH: Isoniazid
RMP: Rifampicin
EMB: Ethambutol
MGIT: Mycobacterial Growth Indicator Tubes
TTD: Time to TB detection
HRCT: High-resolution computed tomography
LAA: Low attenuation area
SD: Standard deviation
CKD: Chronic kidney disease
HREZ: Isoniazid, rifampicin, ethambutol and pyrazinamide.
FEV_1_: Forced expiratory volume in 1 s
MMP: Matrix metalloproteinase
DC: Dendritic cell
CXCL: Chemokine (C-X-C motif) ligand
